# The effect of study-abroad experience on lexical translation among interpreting students

**DOI:** 10.3389/fpsyg.2023.1266921

**Published:** 2023-09-22

**Authors:** Ruiyuan Wang, Jing Han, Bruno Di Biase, Mark Antoniou

**Affiliations:** ^1^School of International Studies, Shaanxi Normal University, Xi'an, Shaanxi, China; ^2^School of Humanities and Communication Arts, Western Sydney University, Sydney, NSW, Australia; ^3^The MARCS Institute for Brain, Behaviour, and Development, Western Sydney University, Sydney, NSW, Australia

**Keywords:** study-abroad experience, Chinese-English, lexical processing, word translation, interpreting students, direction-dependent asymmetry

## Abstract

This study investigates the impact of study-abroad experience (SAE) on lexical translation among 50 Chinese (L1)-English (L2) interpreting students. Participants were divided into two groups based on their experience abroad. Both groups consisted of 25 unbalanced L2 learners who were matched in age, working memory, length of interpreting training, and L2 proficiency. Bidirectional word translation recognition tasks, from L1 to L2 and L2 to L1, highlighted several key findings: (1) both groups were significantly more accurate and faster from L2 to L1 than in the reverse direction; (2) the study abroad (SA) group was more inclined to respond quickly at the risk of making errors, whereas the non-study abroad (NSA) group tended to be more cautious, prioritising accuracy over speed; (3) the SA group were more balanced and consistent in their performance across lexical translations in both directions than the NSA group. These results emphasise the potent effect of SAE in resolving bilinguals’ language competition, especially in streamlining language switching, a cognitive process critical for interpreting students engaging daily with dual languages.

## 1. Introduction

Interpreting, a linguistically complex and cognitively demanding activity, necessitates quick and accurate alternation between two languages within tight temporal constraints (Christoffels et al., 2003). In any interpreting mode, be it simultaneous or consecutive, a lapse or delay in language processing can potentially escalate the cognitive load on interpreters, consequently straining their working memory and affecting their overall interpreting performance (O’Brien et al., 2006). The intricacies of this task underline the paramount importance of efficient lexical retrieval and translation (Mead, 2002; Gile, 2009). Indeed, studies consistently highlight a positive correlation between the speed and accuracy of lexical translation and broader interpreting performance, attesting to the role of lexical processing in interpreting practice (Christoffels et al., 2003; Santilli et al., 2019).

Though some interpreters are often recommended to interpret solely into their L1, many possess the capability for bidirectional interpreting—comprehending in one language and interpreting into another. Yet, it is commonly observed that they may not perform equally well in both directions (Russell and Takeda, 2015). Bilingual individuals often display direction-dependent asymmetry in their lexical processing, with a faster and more accurate performance from their L2 to their L1 than in the reverse direction, indicating an advantage in this direction (Kroll and Stewart, 1994; De Groot and Poot, 1997; Green, 1998; Issa and Shyamala, 2021).

Increasing attention has been paid to factors influencing bilingual lexical translation. Evidence points towards the impact of variables such as participants’ working memory (Sunderman and Kroll, 2009), L2 proficiency (Meuter and Allport, 1999; Costa, 2005; Berghoff et al., 2021; Issa et al., 2022), language use frequency (Christoffels et al., 2007), and language exposure (Kroll et al., 1998; Kroll and Sunderman, 2003; Linck et al., 2008; Kleinman et al., 2022). Among these, the study-abroad experience (SAE) holds significant implications.

SAE, within the field of second language acquisition, is characterised as a type of L2 learning setting that differs from both purely natural exposure and classroom instruction. While natural exposure pertains to the spontaneous, untutored acquisition of a language in its native country, classroom instruction often refers to the teaching of a foreign language in a country where that language is not the primary mode of communication. For instance, one might learn English in Chinese classrooms, with the classroom being the primary, if not the sole, exposure to the language (Muñoz, 2008). SAE, however, integrates formal classroom training with daily life experiences in a country where the target language is dominant, often after students have initially studied the language in their home countries (Xie and Dong, 2021).

Practically, this environment has been shown to increase L2 processing (Antoniou et al., 2015), suppress L1 dominance (Baus et al., 2013), enhance individual cognitive performance (Xie and Dong, 2021), and thus may make it generally easier for bilinguals to access and switch between the two languages (Bonfieni et al., 2019). Theoretically, SAE provides a unique context to examine and challenge existing bilingualism models. It could shed light on the complex interplay of exposure, cognition, and language utilisation in shaping bilingual lexical processing and help refine our understanding of bilingual language control mechanisms.

Most previous research on lexical development in the SAE context mainly concentrates on vocabulary knowledge growth, typically evaluated through word association and word recognition tasks. However, fewer studies have explored how SAE influences a bilingual’s command of two languages, particularly in terms of efficiency (speed and accuracy) and asymmetry in bidirectional lexical translation. Importantly, interpreting students, who consistently keep both languages active even in predominantly monolingual environments, are a unique subgroup of bilinguals (Grosjean, 1997; Babcock and Vallesi, 2017). These students are in a distinct situation as they are training to be professional interpreters, a role that necessitates frequent and skilled language switching. Yet, despite this distinct situation and the demanding requirements of their future profession, few studies specifically investigate the impact of SAE on this group.

Investigating the impact of SAE on interpreting students’ lexical translation performance could offer valuable insights into bilinguals’ lexical processing. Additionally, it may guide interpreting educators in developing effective training tasks to meet specific pedagogical objectives in both study-abroad and home-country classroom settings.

## 2. Literature review

### 2.1. Theoretical framework

Various models have been developed to elucidate the intricacies of bilingual lexical processing. For instance, the Hierarchical Model by Kroll and Stewart (1994) delves into the interrelationships between L1 and L2 words and concepts. In contrast, other models emphasise the lexicon itself (for instance, the role of language nodes in the original BIA model) or advocate for an external mechanism to regulate the lexical system’s operations.

**Figure 1:**
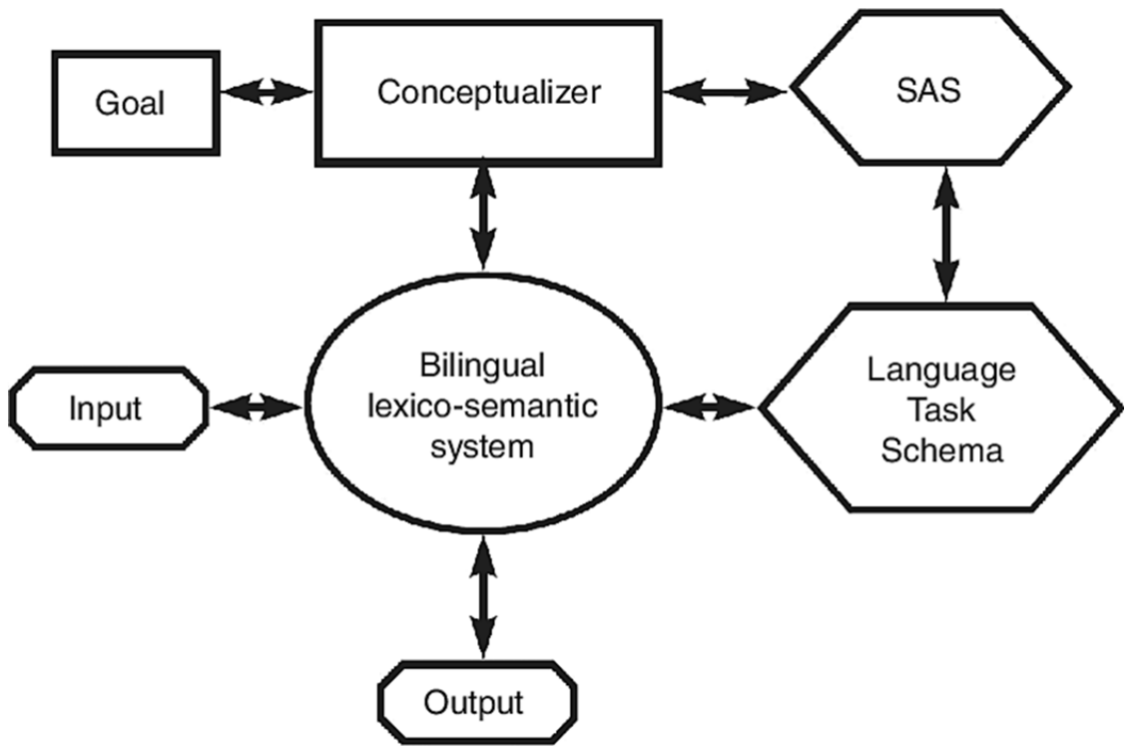
Diagram illustrating the Inhibitory Control model, adapted from Green (1998), showing the interplay of different cognitive processes in language translation.

Our current study predominantly draws upon the Inhibitory Control model introduced by Green (1998). As illustrated in Figure 1. This model is premised on the widely-held linguistic belief that during, or even prior to, speech comprehension and production, elements such as sounds, forms, and concepts from a bilingual’s languages are activated simultaneously in a non-language-specific manner, leading to competition for selection (Odlin, 2003, 2012; Jarvis and Pavlenko, 2008).

The Inhibitory Control model postulates that the selection of the target language at any given time involves the suppression of the non-target one. This suppression process is overseen by the Supervisory Attentional System (SAS), a superior cognitive control mechanism that intervenes with the language system as needed. One of the key insights derived from the model is the relationship between language proficiency and inhibition. The more proficient a bilingual is in a language, the more effortlessly they can suppress the non-target language, and conversely, lesser proficiency might entail greater cognitive effort in this suppression (Meuter and Allport, 1999; Meuter, 2009). Inherent to this bilingual ability is the concept of ‘switching costs’, which are cognitive tolls associated with toggling between languages. ‘Direction-dependent asymmetry’ epitomises these costs. Given that the dominant L1 generally has a heightened activation compared to the less dominant L2, more cognitive resources are required to inhibit L1, thus facilitating L2 production. In essence, bilinguals often exhibit a faster, more accurate performance when switching from their L2 to their L1 compared to the reverse direction (Kroll and Stewart, 1994; De Groot and Poot, 1997; Green, 1998; Issa and Shyamala, 2021). With this intricate interplay between bilingualism and cognitive functions in mind, it becomes imperative to explore how immersive bilingual experiences, such as SAE, further influence this dynamic.

### 2.2. Impact of study-abroad experience on bilinguals’ cognitive performance

Lexical translation extends beyond a mere linguistic task; it is intrinsically woven with cognitive processes, especially those governed by working memory. As a fundamental pillar of cognitive resources, working memory is predictive of a host of intricate cognitive tasks, inclusive of language processing (Daneman and Merikle, 1996; Miyake and Friedman, 1998; Ardila, 2003). At its core, working memory boasts an executive function, acting as a cognitive controller (Baddeley, 1996). This controller is responsible for tasks such as selective attention, distraction inhibition, and overall coordination (Baddeley, 1996, 2017). Study-abroad experience, with its intensive L2 immersion, has the potential not just to mould lexical proficiency but also to influence the very cognitive mechanisms that underpin bilingual language processing (DeLuca et al., 2020).

Researchers suggest that the regular, habitual use of the bilingual control mechanism to reconcile the L1 and L2 competition should have cognitive benefits (Ransdell et al., 2006; Linck et al., 2008; Bialystok et al., 2009; Bartolotti et al., 2011; Xie and Dong, 2017). Owing to the constant regulation of two language systems as a result of SAE, bilinguals in this language environment are exposed to extensive practice of executive functions of language control on daily basis, which reduces the dominance of L1 and makes it generally easier to access and switch between the two languages (Bonfieni et al., 2019; Xie and Dong, 2021).

A substantial body of evidence indicates that bilingual cognitive control capabilities can be moulded by diverse bilingual experiences and language use frequency (e.g., Costa et al., 2009; Prior and Gollan, 2011; Xie and Dong, 2017; Xie and Dong, 2021). For instance, Xie and Dong (2017), provided empirical support for the assertion that public speaking training experience can enhance bilinguals’ cognitive control capabilities. Furthering this line of research, Xie and Dong (2021) examined the potential cognitive control disparities between bilinguals with SAE and those without. Despite matching both groups based on demographic factors like age, socioeconomic status, and intelligence, the study found that SAE was associated with a significant advantage in cognitive control, particularly in mental set shifting. This enhancement was attributed to increased L2 usage and switching within the SAE context. As SAE seemingly enhances cognitive mechanisms through frequent L2 use, it’s plausible to inquire how this immersive experience specifically impacts lexical processing, an integral part of bilingual language utilisation.

### 2.3. Impact of study-abroad experience on lexical processing

Despite the prevalence of SAE in L2 learning programmes, research examining the effects of SAE on lexical processing remains limited and somewhat inconclusive (Hamano-Bunce et al., 2019). The majority of prior research has focused on the impact of SAE on L2 receptive vocabulary knowledge growth (e.g., Fitzpatrick, 2012; Issa et al., 2020; Kleinman et al., 2022), or L2 productive vocabulary development (Segalowitz and Freed, 2004; Llanes and Muñoz, 2009; Barquin, 2012; Pérez-Vidal et al., 2012; Lara, 2014), or its contribution to enhanced sensitivity to L2 speech (Grey et al., 2015). However, a closer examination reveals that the cognitive advantages facilitated by SAE have profound implications on lexical processing.

For instance, within the realm of SAE and L2 lexical processing, Chinese speakers who spent an average of 6.5 years in the US demonstrated better performance in their speed and accuracy in recognising spoken L2 words. This suggests that SAE participants may possess a heightened capability to decipher talker variability and expedite nonnative language processing with fewer cognitive resources than their counterparts without such experience (Antoniou et al., 2015).

The avenue of research addressing SAE’s influence on the decline of L1 availability has also yielded significant insights. Notably, studies have indicated that extended immersion in an L2 context amplifies the cognitive effort required for languages that are not regularly practised or used (Tu et al., 2015). A substantial body of research, often focusing on typologically similar language pairs like English-Spanish, has showcased a decline in L1 lexical representation during immersion in L2 settings (Linck et al., 2009; Kaushanskaya et al., 2011; Baus et al., 2013).

On another front, SAE also appears to shape bilinguals’ communication behaviours. A study by Tokowicz et al. (2004) observed that individuals with SAE experience often ventured answers even when they were uncertain about accurate word translations, suggesting a bolstered propensity to communicate irrespective of potential inaccuracies.

Collectively, these studies highlight the intricate interplay between the cognitive mechanisms honed through SAE and their subsequent manifestation in bilingual lexical processing.

### 2.4. Research gaps and aims

Fundamental questions remain regarding the impact of SAE on lexical translation. Notably, studies on Chinese-English language pairs, particularly with Chinese as L1 and English as L2, are significantly underrepresented—this is significant given the rising trend of Chinese students studying abroad in English-speaking countries (Bhandari, 2017; Pavlacic, 2018; Szego, 2020). The unique linguistic characteristics of interpreting students due to their constant activation of both languages have been largely sidelined in SAE research. Such linguistic training may interact with SAE in ways that either mitigate or amplify its impact. Furthermore, the relationship between bidirectional lexical translation and SAE has been sparsely explored. Previous studies have often examined the impact of SAE on either L1 or L2 lexical processing, neglecting the potential influence of SAE on the bidirectional lexical translation efficiency and asymmetry.

In light of these identified gaps, our study seeks to determine the effect of SAE on bidirectional word translation tasks among interpreting students with Chinese as L1 and English as L2. Specifically, we examine how SAE impacts their efficiency (measured by accuracy and response times) and direction-dependent asymmetry in bidirectional translation.

Drawing from the literature that highlights the benefits of SAE, we hypothesise that participants with SAE in English-speaking countries will demonstrate better efficiency and balance in bidirectional word translation tasks compared to those without such experience.

## 3. Materials and methods

### 3.1. Participants

To ensure a homogenous sample, we only included participants who shared a similar linguistic and cultural background. All participants were native Chinese speakers, aged between 20 and 30, a criterion chosen based on existing research indicating the influence of age and cultural background on brain activity (Signorelli et al., 2012; Han and Ma, 2014).

As detailed in Table 1, the study comprised 50 participants, all of whom were pursuing master’s degrees in Chinese-English bidirectional interpreting and translation. They were all in their third semesters and had commenced mandatory English education at the age of 12, following the establishment of their L1. Participants were evenly divided into two groups based on their SAE.

**Table 1:** Descriptive information of participant groups (mean and standard deviation).

Characteristic	SA group	NSA group
Number of participants	25.0	25.0
Age (years)	25.0 (0.31)	24.0 (1.19)
Years of study abroad	3.64 (0.46)	0 (0)
University semester of interpreting learning	3.0 (0)	3.0 (0)
Age of onset (learning L2)	12.1 (0.65)	12.1 (0.73)
L2 Interacting with English speakers	37.5** (2.04)	19.9** (1.40)
Watching TV	25.0 (0.96)	27.7 (1.87)
Reading	32.0 (1.36)	31.9 (1.91)
Self-instruction	26.4 (0.71)	26.4 (0.68)

*p < 0.05, **p < 0.01, and ***p < 0.001.

The study abroad (SA) group, consisted of 25 students, each enrolled in one of these three universities in Sydney, Australia (nine from Western Sydney University, eight from Macquarie University and eight from the University of New South Wales). They had an average SAE duration of 3.64 years and an average age of 25. In their Australian academic environment, they typically engaged in interpreting and translation classes conducted mainly in English for 25–30 h weekly. While interactions with faculty and international peers were predominantly in English, they reverted to Chinese for conversations with fellow Chinese students.

On the other hand, the non-study abroad (NSA) group was composed of 25 students from three universities in Xi’an, mainland China (seven from Shaanxi Normal University, eight from Xi’an Jiaotong University and 10 from Xi’an International Studies University). These students had not ventured to English-speaking nations and had an average age of 24. Their curriculum consisted of approximately 28–30 h of interpreting and translation weekly. However, the teaching methodology leaned heavily on their native Chinese language, and their daily interactions seldom involved English.

To further elucidate participants’ linguistic profiles, we utilised the Language Experience and Proficiency Questionnaire (LEAP-Q). Widely recognised in linguistic and psycholinguistic research (e.g., Bialystok et al., 2009), this tool provided insights into participants’ weekly L2 exposure. Notably, the SA group reported significantly higher weekly interaction hours with English speakers than the NSA group, though other linguistic activities remained comparable for both groups.

### 3.2. Materials

#### 3.2.1. Working memory span

Participants’ working memory resource availability was assessed using the English reading span task (Daneman and Carpenter, 1980), administered via E-Prime Professional 2.0. Sentences were displayed on the screen one at a time, and participants were instructed to read each sentence aloud, evaluate its semantic plausibility, and attempt to remember the final word of each sentence. Sentences were presented in sets of increasing size, ranging from two to five sentences. There were three series for each set size, resulting in a total of 42 sentences. Each sentence contained 11 to 13 words and concluded with a distinct word. Half of the sentences in this task were semantically plausible, while the remaining half were implausible.

Upon completion of each set, participants were prompted to recall as many sentence-final words as they could. No restrictions were imposed on the order or duration of the recall. Participants’ recalled final words were considered valid only if accompanied by accurate judgements regarding sentence plausibility. The number of correctly recalled final words served as an indicator of a participant’s working memory span.

#### 3.2.2. L2 proficiency

This study emphasises L2 proficiency due to the background of our participants. Being native Chinese speakers, their proficiency in their L1 is uniformly high and consistent for daily communication. In contrast, as English is their foreign language, their L2 proficiency is expected to vary. They were asked to complete LexTALE, a reliable and standardised online test of general English proficiency that is widely employed in linguistic studies (e.g., Christoffels et al., 2007; Lemhöfer and Broersma, 2012; De Bruin et al., 2014; Keuleers et al., 2015). This assessment required participants to determine whether a given sequence of letters displayed on the screen constituted an English word. Upon completion of the task, the participants’ scores were immediately calculated and displayed on the screen.

#### 3.2.3. Bidirectional word translation

Participants’ lexical processing performances were assessed using word translation recognition tasks, administered via E-Prime Professional 2.0 and Chronos. Distinct versions of the word translation recognition task were employed for both language directions (English-Chinese and Chinese-English). The stimuli consisted of 60 English and 60 Chinese words, which were presented auditorily in a randomised order for each participant using E-Prime Professional 2.0. During each trial, participants listened to a word through headphones, and the correct translation equivalent or an incorrect, misleading word was displayed on the screen. Participants were instructed to rapidly and accurately identify whether the word on the screen corresponded to the correct translation of the auditorily presented word by pressing the Yes or No buttons on the Chronos box. No time limit was imposed on participants’ responses.

Two word characteristics—word frequency and word length—were carefully controlled across stimuli and target translation equivalents in both translation directions (L1-L2 and L2-L1) to ensure the generalisability of our findings.

Chinese and English word frequencies were obtained from the SUBTLEX-UK (Van Heuven et al., 2014) and SUBTLEX-CH (Cai and Brysbaert, 2010) word frequency databases, respectively. As demonstrated in Table 2, Chinese and English words used in the task were matched for word frequency. This similarity of frequency and word length indicates that any differences in accuracy or response time during task performance are not attributable to disparities in word frequency between the misleading words and target translation equivalents, but rather are a true reflection of participants’ lexical translation performance.

**Table 2:** Mean word frequency and length of stimuli (mean and standard deviation).

Word	Directions	Appear form	Frequency (per million)	Word length
Chinese	E-C	Target Chinese translation	3.6 (1.04)	2 (0)
		Written misleading Chinese	3.6 (1.16)	2 (0)
	C-E	Audio presented Chinese	3.5 (0.93)	2 (0)
English	E-C	Audio presented English	3.6 (0.90)	7 (1.61)
	C-E	Target English translation	3.5 (0.90)	7 (1.81)
		Written misleading English	3.2 (0.40)	7 (1.86)

Nonetheless, matching word length across Chinese and English is exceedingly challenging, given their typological distinctions as languages. This study only ensured that the Chinese words employed in both directions comprised a comparable number of characters, while the lengths of the English words utilised in the task were similarly consistent.

Participants’ accuracy (%) and response times (ms) were calculated, with only the response times of accurate trials incorporated into the data analysis. Although translation has long been a conventional pedagogical task in L2 and interpreting training, employing a response time-based translation task as a research instrument remains relatively novel (Issa and Shyamala, 2021).

#### 3.2.4. Post-task interview

To delve deeper into participants’ subjective experiences and cognitive reflections on lexical translation performance, we conducted a post-task interview. This complementary approach aimed to understand both their self-evaluations of the lexical translation and the underlying reasons for their perceptions.

The interview served two primary purposes:

Firstly, we provided a quantitative self-assessment, allowing participants to rate their performance on a five-point scale (1 = very poor, 5 = excellent).

Secondly, we sought qualitative insights through an open-ended question. This was designed to reveal the underlying factors or thought processes influencing their ratings, providing a deeper understanding of their performance beyond the quantitative assessment.

All interviews were conducted in their native language to ensure comfort and clarity in communication.

### 3.3. Procedure

Participants consented to the research conducted in a quiet university space using a Lenovo laptop and E-Prime Professional 2.0 software. All verbal instructions were delivered in their native language. Tasks involved word translation recognition and reading span, with response times and accuracy recorded via a Chronos response box. The task order was counterbalanced to mitigate fatigue effects. The session, approximately 30 min, was audio-recorded. It included consent, LexTALE completion, memory span task, bidirectional word translation tasks, and a post-task interview.

## 4. Results

Table 3 shows the descriptive analysis of participants’ working memory spans and L2 proficiency, skewness and kurtosis indicate that the means are normally distributed.

**Table 3:** Descriptive analysis of study abroad (SA) and non-study abroad (NSA) groups’ working memory spans and L2 proficiency (mean and standard deviation).

	SA	NSA
Working memory	25.0 (5.90)	24.4 (5.46)
L2 Proficiency	63.1 (7.91)	65.5 (10.73)

Two groups were comparable in their mean working memory, *t*(24) = 0.30, *p* = 0.77. and their L2 proficiency assessed by LexTALE, *t*(24) = 0.85, *p* = 0.41.

### 4.1. Overall direction-dependent asymmetry in accuracy

**Figure 2:**
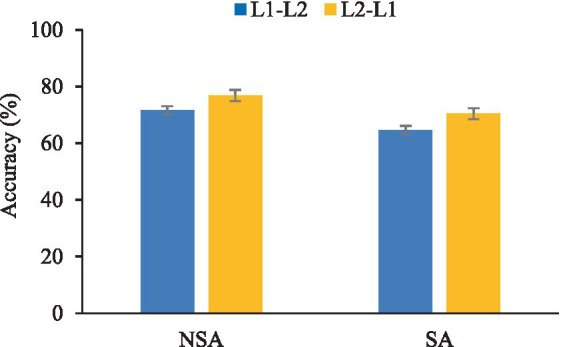
Bar graph showing the overall word translation recognition accuracy (in percentage) in Chinese-English (L1-L2) and English-Chinese (L2-L1) directions across both groups regardless of language environment.

The impact of SAE on the groups’ bidirectional word translation was explored via a 2 × (2) mixed factorial ANOVA with the within-subjects factor of the direction of translation (L2-L1 vs. L1-L2) and the between-subjects factor of the group (NSA vs. SA). A significant main effect of direction was observed in accuracy, *F*(1, 48) = 35.02, *p* < 0.001, $\eta_{p}^{2}$ = 0.422, as depicted in Table 4 and Figure 2. This indicated that both groups exhibited the same direction-dependent asymmetry: word translation from the L2-L1 direction was more accurate than in the opposite direction.

**Table 4:** Word recognition accuracy from Chinese to English (L1-L2) and English to Chinese (L2-L1; mean and standard deviation).

	SA	NSA
L1-L2 (% correct)	65% (0.08)	72% (0.08)
L2-L1 (% correct)	70% (0.10)	77% (0.10)

#### 4.1.1. Comparison of study abroad and non-study abroad groups’ accuracy

According to the 2 × (2) mixed factorial ANOVA, a significant main effect of group was observed in the two groups’ accuracy, *F*(1, 48) = 8.51, *p* = 0.005, $\eta_{p}^{2}$ = 0.151, which suggests that the NSA group is more accurate than the SA group in retrieving words in both directions (mean accuracy rate: 74% vs. 68%, respectively).

#### 4.1.2. Interaction between group accuracy and translation direction

According to the 2 × (2) mixed factorial ANOVA, the group × direction interaction was not significant in both groups’ accuracy, *F*(1, 48) = 0.05, *p* = 0.83, $\eta_{p}^{2}$ = 0.001. This finding suggests that there is no statistically significant difference in the interaction between language direction (L1-L2 or L2-L1) and lexical translation accuracy among the two groups (SA and NSA). In more precise terms, SAE does not result in a discernible variation in the accuracy with which students execute lexical translation tasks across language directions in comparison to their counterparts without such experience.

### 4.2. Overall direction-dependent asymmetry in response times

**Figure 3:**
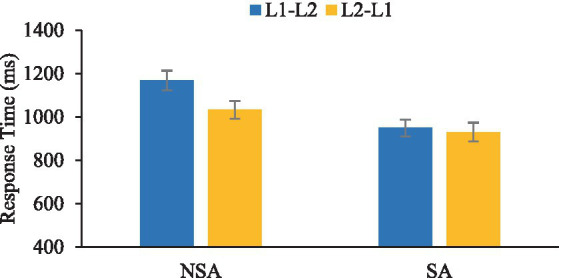
Graph depicting the overall word translation recognition response times (in milliseconds) in Chinese-English (L1-L2) and English-Chinese (L2-L1) directions for both groups.

The impact of SAE on the groups’ bidirectional word translation was explored via a 2 × (2) mixed factorial ANOVA with the within-subjects factor of the direction of translation (L2-L1 vs. L1-L2) and the between-subjects factor of the group (NSA vs. SA). A significant main effect of direction was also observed in response times, F(1, 48) = 8.96, *p* = 0.004, $\eta_{p}^{2}$ = 0.157, as depicted in Table 5 and Figure 3. This indicated that both groups exhibited the same direction-dependent asymmetry: word translation from the L2-L1 direction was more efficient than in the opposite direction.

**Table 5:** Word recognition reaction times from Chinese to English (L1-L2) and English to Chinese (L2-L1; mean and standard deviation).

	SA	NSA
L1-L2 (ms)	948.7 (193.95)	1167.9 (193.95)
L2-L1 (ms)	929.9 (214.25)	1032.5 (205.76)

#### 4.2.1. Comparison of study abroad and non-study abroad groups’ response times

According to the 2 × (2) mixed factorial ANOVA, a significant main effect of group was observed in response times, F(1, 48) = 9.05, *p* = 0.004, $\eta_{p}^{2}$ = 0.159. This main effect indicates that the SA group demonstrates faster response times than the NSA group in accurately retrieving words in both directions (939.3 ms vs. 1,100.2 ms, respectively). This is evidenced by the shorter response times in the SA group. Given that the SA group exhibited faster response times yet lower accuracy than the NSA group, this phenomenon may be indicative of a speed-accuracy trade-off.

#### 4.2.2. Interaction between group response times and translation direction

According to the 2 × (2) mixed factorial ANOVA, a significant group × direction interaction was observed in response times, *F*(1, 48) = 5.134, *p* = 0.028, $\eta_{p}^{2}$ = 0.097. To further investigate this interaction, the authors conducted pairwise comparisons using an adjusted alpha level of 0.025. The NSA group exhibited a significant difference in their word translation recognition response times for L1-L2 vs. L2-L1, *t*(24) = 4.17, *p* < 0.001. In contrast, no statistical difference was detected for the SA group, *t*(24) = 0.47, *p* = 0.64, which suggests a more balanced performance across the two language directions among the SA group.

#### 4.2.3. Post-task interview

Table 6 presents the self-rating results of the word translation recognition task. No significant difference was observed between the SA and NSA groups, *t*(24) = 0.65, *p* = 0.52. This indicates that both groups perceived their performance as somewhere around ‘average’.

**Table 6:** Participants’ self-rating on word translation recognition performance.

	SA	NSA
Self-rating	3.16 (0.62)	3.04 (0.73)

Delving into the qualitative feedback from the open-ended question, a noteworthy pattern emerged. A substantial 17 out of 25 SA participants felt they had rushed through the task. They expressed a post-submission realisation of their errors, indicating a premature commitment to answers. One SA participant reported, ‘Although I thought the task would be challenging at first, I later found it to be less difficult than I had anticipated. Nevertheless, I still felt compelled to respond as quickly as possible, maybe due to some external pressure or personal drive’. In contrast, only 2 out of 25 NSA participants reported similar experiences, with most commenting on the difficulty of the task, such as confusing distractors that resembled the correct words.

## 5. Discussion

### 5.1. Descriptive analysis of working memory and L2 proficiency

Based on the results presented in Table 3, there appears to be no significant difference in two working memory and L2 proficiency between the SA and NSA groups. This suggests that the two groups are comparable in terms of these cognitive and linguistic measures, ensuring that any differences observed in other measures are less likely to be attributed to discrepancies in working memory or baseline L2 proficiency.

### 5.2. Overall direction-dependent asymmetry in accuracy and response times

As illustrated in Table 4 and Figure 2, both groups’ word translation from the L2-L1 direction was more accurate and faster than in the opposite direction. This result replicates previous research findings that L1-L2 lexical translation is slower and more error-prone than L2-L1 (e.g., Meuter and Allport, 1999; Costa and Santesteban, 2004; Linck et al., 2008; Meuter, 2009; Peeters et al., 2014; Olson, 2017). The finding also lends support to the Inhibitory Control model (Green, 1998), which argues that in unbalanced bilinguals, the dominant L1 is more active, requiring greater cognitive effort to suppress during language processing. The model states that L1-L2 word translation entails inhibiting the dominating L1 competitors to ensure that the intended L2 words are chosen for output while both languages are activated in a non-selective way (see Green, 1998; Kroll et al., 2008). L1-L2 direction thus demands more mental effort, realised as lower accuracy and longer response times, than suppressing the comparatively weaker L2 in the opposite translation direction.

What is noteworthy is the consistency of this direction-dependent asymmetry across both groups, regardless of their SAE. This consistency echoes findings by Meuter and Allport (1999), which proposed that bilinguals’ translational asymmetry is primarily influenced by the proficiency of their languages. Even though SAE contributes to inhibiting L1 dominance and facilitating L2 processing in the current study, it appears that for our group of late unbalanced bilinguals, a three-year SAE was insufficient to counteract L1 dominance. Additionally, all our participants were interpreting students who routinely switch between two languages. This habitual language-switching may have counteracted the attenuation impact on their L1 in the SAE. Consequently, both SA and NSA groups demonstrated the same direction-dependent asymmetry in their word translation recognition tasks. These findings underscore the intricate interplay of language proficiency, immersion, and cognitive control in bilingual contexts.

### 5.3. Group differences in accuracy and response times

A key differentiator between the two groups in this study is the SAE. The SA group demonstrated faster response times but lower accuracy in lexical translation compared to the NSA group, suggesting a speed-accuracy trade-off. To shed light on these observed differences, we integrated insights from post-task interviews and LEAP-Q results.

#### 5.3.1. Post-task interview insights

Though self-ratings indicated similar self-perceptions of performance across both groups (Table 6), qualitative feedback highlighted differing lexical translation approaches. Notably, a significant portion of the SA group felt they had rushed through the task and later realised their mistakes indicating a potential inclination towards prioritising speed over accuracy. This inclination could be influenced by their experiences in real-life scenarios where rapid responses were essential. This focus on speed was further echoed in remarks from SA participants, underscoring the potential conditioning effect of real-time English interactions.

#### 5.3.2. Language experience and proficiency questionnaire results

The SA group engaged more frequently with English speakers on a weekly basis than the NSA group. Given that regular L2 usage can bolster learners’ communicative willingness (Dewaele, 2019), SA participants may exhibit a reduced stringency when selecting translation equivalents, potentially sacrificing precision. Conversely, NSA participants appeared more methodical, prioritising accuracy and delving deeper into the task’s challenges.

Our observations align with research by DeKeyser (1991) and Tokowicz et al. (2004), which emphasised SAE’s impact on lexical translation accuracy. They noted that individuals with extended SAE (exceeding 1 year) were more likely to guess unknown word meanings, despite error risks, contrasting with those with limited or no SAE.

The potential role of cognitive control also merits attention. Previous research highlights the intricate relationship between language use experience and cognitive control abilities (Green and Abutalebi, 2013). Xie and Dong (2017) found that individuals with public-speaking training experience exhibited quicker response times than both monolinguals and a control bilingual group. Similarly, continuous L2 engagement, characterised by intensive semantic, attentional demands, and unwanted behaviour or word suppression, could potentially sharpen cognitive control, as found in studies on public speakers. Our SA group’s frequent English interactions could be seen as a catalyst for their enhanced cognitive control, which might manifest in swifter response times.

It is crucial to differentiate between working memory and cognitive control. While working memory manages information retention and manipulation, essential for tasks like language processing (Baddeley, 2017), cognitive control oversees and coordinates cognitive processes, especially amidst distractions. This includes functions such as attentional focus, inhibition, and task switching, vital in bilingual contexts.

In this study’s context, both SA and NSA groups having similar working memory capacities suggests equivalent foundational cognitive capabilities. Yet, differences in their SAE could have uniquely moulded their cognitive control faculties. Regular immersion in L2, as experienced by the SA group, intensifies cognitive control demands due to continuous language switching, L1 inhibition, and tackling L2 challenges. Such immersion could have finetuned the SA group’s cognitive control, even if their working memory remained consistent with the NSA group.

This variance may explain the observed differences in cognitive control between the two groups, despite comparable working memory capacities. It is not a claim of cognitive superiority but indicates that linguistic experiences might influence cognition differently. Future studies should delve deeper into this by evaluating working memory and cognitive control in comparable bilingual cohorts.

### 5.4. Interaction between group and translation direction

The interaction effects in the context of accuracy and response times yielded contrasting insights. While there was no interaction effect for accuracy (indicating that both groups showed a similar pattern of direction-dependent asymmetry in accuracy), there was a significant interaction for response times. The NSA group displayed a notable difference in their response times for L1-L2 vs. L2-L1, whereas the SA group did not.

These findings imply that having an SAE might lead to a more consistent and balanced performance in word translation across both language directions. In contrast, those without such an experience may face more variability in their translation speeds depending on the direction.

Our findings replicate that of Schwartz and Kroll (2006) and Chmiel (2016) that the SA group was more balanced and consistent in terms of lexical processing during word translation than the NSA group. Previous studies have suggested that factors such as participants’ L2 proficiency (Meuter and Allport, 1999; Costa and Santesteban, 2004; Costa, 2005), language-switching habits (Christoffels et al., 2007), working memory (Sunderman and Kroll, 2009), and learning contexts (Kroll et al., 1998; Kroll and Sunderman, 2003; Linck et al., 2008) impact on the language inhibitory process, and influence the efforts involved in lexical processing. In the present study, participants in the SA and NSA groups were all interpreting students, and therefore, all were engaged in bilingual processing on a daily basis. Moreover, they were also comparable in their L2 proficiency, word knowledge and working memory resource availability. Therefore, the smaller degree of asymmetry observed in the SA group may be attributed to their habitual toggling between two languages during SAE.

For the SA group, immersion in an L2-rich environment granted them a distinct advantage by allowing them to suppress interference from their dominant L1 more effectively (e.g., Linck et al., 2009; Baus et al., 2013). This reduced effort in inhibiting the L1 during L2 processing manifested even though their L1 remained dominant. Consequently, the SA group exhibited more consistent and less asymmetric performance in bilingual processing compared to their NSA counterparts.

It is well-established that prolonged exposure to bilingual environments sharpens a bilingual’s ability to switch languages (e.g., Bialystok and Barac, 2012; Nicolay and Poncelet, 2015). Moreover, frequent daily engagements with L2, as observed in study-abroad bilinguals, not only diminish the influence of L1 but also enhance the ease of transitioning between both languages (Bonfieni et al., 2019). Echoing Xie and Dong (2021), such bilinguals predominantly engage in English, particularly when interacting with peers, making linguistic toggling commonplace. This frequent language transition, characteristic of the SA group, underlines their improved mental set shifting, leading to a more balanced bilingual lexical translation compared to their NSA counterparts.

In contrast, for NSA participants in our study, their daily linguistic environment was predominantly aligned with their native L1. This might have heightened the challenge of suppressing the ever-present L1 during L2 processing, thus skewing their bilingual lexical translation. Moreover, their interactions in English were notably fewer than those of the SA group, as indicated by their LEAP-Q results. This suggests that they predominantly communicated in Chinese, especially with fellow Chinese students, limiting their opportunities for smooth transitions between languages. The absence of a consistent L2-rich environment might deprive the NSA group of the routine that aids mental set shifting, especially the transition between languages. Consequently, this might lead to a pronounced degree of asymmetry in the NSA group’s performance.

Furthermore, it is pertinent to underscore the unique linguistic profile of interpreting students. As trainees navigating two languages on a daily basis, they embody a distinct category of bilinguals. Language switching is integral to their training, sharpening their ability to transition between languages rapidly. Even amidst this frequent toggling, the impact of SAE on lexical processing emerged prominently in our findings. Such a pronounced effect, in spite of their rigorous linguistic exercises as interpreting students, further underscores the profound influence of SAE. It suggests that while routine interpreting practices equip students with certain bilingual proficiencies and cognitive advantages, immersion in an authentic language environment through SAE offers unparalleled benefits.

To wrap up our discussion, our findings indicate that concerning direction-dependent asymmetry, both SA and NSA groups were more adept in translating words from L2 to L1 than vice versa. Although we postulated that the SA group showcased more efficient bidirectional word translation, the results were multifaceted. While the SA participants were faster across translation directions, the NSA group exhibited greater accuracy. This divergence hints at a speed-accuracy trade-off: SA participants, due to their extensive L2 usage and frequent language toggling, might prioritise speed and a propensity to communicate, even without the precise word.

Moreover, our research underlines that SAE aids in achieving a less asymmetric performance in word translations across languages. Such observations emphasise the instrumental role of SAE in alleviating language competition, diminishing the cognitive strain tied to bilingual lexical processing, and fine-tuning the cognitive mechanism managing bilingualism.

This study is not without its limitations, notably its cross-sectional design. Adopting a longitudinal methodology, tracking the same group of students during their SAE might offer richer insights into the progression of their lexical translation performance.

Additionally, we assessed only the overarching working memory resources, bypassing specific facets of cognitive control. Prospective research should delve into this, appraising both working memory and cognitive control in comparable bilingual groups.

Lastly, the SAE is a composite of linguistic immersion and the intricacies of residing in a foreign country. While our participants’ increased willingness to communicate might arise from intensified linguistic exposure and interaction in a study-abroad context, it could also be shaped by non-linguistic elements like the process of cultural adaptation. As Xie and Dong (2021) have highlighted, the confluence of these components—linguistic immersion, cultural adjustments, and other unique challenges faced abroad—might collectively influence bilingual performance and communicative behaviour. This complex interplay undeniably warrants further exploration.

## 6. Conclusion

This study elucidates the impact of SAE on bidirectional lexical translation among Chinese (L1) English interpreting students. While previous research has touched upon the effects of SAE on bilingual translation, our findings augment this body of knowledge by highlighting the performance differences between the SA and NSA groups. Notably, the SA group showcased superior consistency in their translations and displayed heightened communicative willingness.

Recognising the crucial role of lexical processing in higher-order language processing, including interpreting, there’s an evident need for pedagogical adjustments. We advocate for universities to bolster communicative activities both within and beyond the curriculum, thereby immersing students more deeply in their L2. Such active engagement can potentiate the activation of their L2, mitigating the cognitive burdens of L2 processing and language switching. This approach bears significant relevance for interpreting learners, aiding them in honing critical skills for their academic and future professional endeavours.

While numerous studies have delved into bilingualism across varied language-learning contexts, there remains a paucity of research focusing on the SAE’s impact on the Chinese-English language pairs, especially among interpreting students. As such, our results not only bridge this gap but also furnish actionable insights for L2 educators and interpreting trainers. The findings are especially pertinent for interpreting students without the advantage of SAE, offering them strategies to compensate for their limited interactions in L2 environments.

## Data availability statement

The raw data supporting the conclusions of this article will be made available by the authors, without undue reservation.

## Ethics statement

The studies involving humans were approved and conducted by Western Sydney University Human Research Ethics Committee’s guidelines (approval number H12405). The studies were conducted in accordance with the local legislation and institutional requirements. The participants provided their written informed consent to participate in this study.

## Author contributions

RW: Conceptualization, Data curation, Formal Analysis, Investigation, Writing – original draft, Writing – review & editing. JH: Supervision, Writing – review & editing. BB: Supervision, Writing – review & editing. MA: Data curation, Formal Analysis, Software, Supervision, Writing – review & editing.
